# Tobacco/Isatis intercropping system improves soil quality and increase total production value

**DOI:** 10.3389/fpls.2024.1458342

**Published:** 2024-11-18

**Authors:** Zhongyan Wang, Xiaomeng Guo, Shoutao Cao, Mingfeng Yang, Qiang Gao, Hao Zong, Xianchao Shang, Yun Gao, Li Zhang, Long Yang, Miao Zhang, Mingming Sun, Xin Hou

**Affiliations:** ^1^ College of Plant Protection, Shandong Agricultural University, Tai’an, China; ^2^ China Tobacco Shandong Industrial Co., Ltd., Qingdao, Shandong, China; ^3^ Shandong Corporation of China National Tobacco Company, Jinan, Shandong, China

**Keywords:** intercropping, tobacco, Isatis, bacterial community, soil microbe

## Abstract

Continuous tobacco monocropping has caused soil degradation and yield reduction in China. Intercropping, as a specific and efficient cropping pattern, is highly associated with the enhancement of soil quality and land-use efficiency. Tobacco (*Nicotiana tabacum* L.)- Isatis (*Isatis tinctoria* L.) intercropping can significantly alleviate pests and diseases, and improve soil potential and fertility in tobacco fields. In this study, comparative analysis of three different tobacco-Isatis intercropping (B2, B3, B4) and tobacco monocropping (B1) on the soil nutrients, enzyme activities, and microbial community were conducted. B2, B3, and B4 importantly increased the contents of organic matter, available potassium, and available phosphorus content of the soil by 17.38%, 7.76%, and 2.78%, respectively. Moreover, B2 enhanced the activities of sucrase, urease, and catalase of soil by 2.35 times, 3.16 times, and 4.47 times, respectively, and B3 enhanced the activities of sucrase, urease, and catalase of soil by 2.74 times, 3.22 times, and 3.11 times, respectively. The intercropping pattern also optimized the structure of the soil microbial community. The relative abundances of Acidobacteriota, Chloroflexi, Gemmatimonadota, Planctomycetota, Nitrospirota, and Verrucomicrobiota in B3 and B4 were higher than those in B1. Positive links in soil bacterial correlation networks accounted for 47%, and soil bacteria formed a highly interactive and complex network. And compared with the B1, Ascomycota and Basidiomycota were lower abundance in B2 and B4, Ascomycota were lower abundance in B3 and Mortierellomycota were lower abundance in B2 and B3. Compared with monocropping, the chemical composition of tobacco leaves was harmoniously improved and the total production value of tobacco fields was significantly higher. The content of reducing sugar, total sugar, nicotine, potassium, and two-sugar ratio of leaves were increased after intercropping. The proportion of top-grade tobacco leaves after roasting in B2, B3, and B4 treatments were increased by 8.19%, 16.74%, and 27.32%, respectively. The study constructs insights into microbial community interactions at in tobacco/Isatis intercropping systems, and may facilitate the further development of tobacco/Isatis intercropping systems.

## 1 Introduction

Tobacco is an extremely vital cash crop and the main sources of tax revenue in China. As the quality of human life increases, the area of land available for cultivation continues to decrease, monocropping has been the main planting form in agriculture (Gao et al., 2019). Undoubtedly, successive years of cultivation has caused many problems in production, such as low yields, nutritional disorders in oilseeds and the emergence of diseases, which have wreaked havoc on agricultural systems (Ding et al., 2021; Jain et al., 2021). A three-year continuous crop showed negative effects on the agronomic traits of tobacco plants, such as plant height and stem circumference. Continuous cropping obstacle has become a worldwide problem in tobacco production. Intercropping is a traditional planting method, which can avoid wasting planted area and effectively improves the planting quality (Wang et al., 2021; Nasar et al., 2022).

There has been much research about the impact of intercropping (Weerarathne et al., 2017; Shen et al., 2018). A reasonable intercropping system considerably improved soil structure, repaired soil environment, and effectively controlled continuous cropping obstacles (Rosenzweig et al., 2018). Intercropping played vital roles in protecting against soil-borne diseases such as tobacco black shank (Ding et al., 2021), tomato bacterial wilt (Lai et al., 2011; Tan et al., 2021), faba bean fusarium wilt (Zheng et al., 2022), soybean red crown rot (Gao et al., 2014), peanut root rot (Li et al., 2014). For instance, faba bean intercropping with wheat effectively controlled benzoic acid damage by controlling related genes, thereby reducing related pests and diseases (Zheng et al., 2023). Xiao et al. exported that intercropping of maize with sesame, peanut, soybean, and sweet potato significantly improved ecological functions and increased the number of bacterial and fungal species in the soil (Xiao et al., 2023). Additionally, Tobacco-rosemary intercropping was found to activate effective phosphorus, while intercropping tobacco with aromatic plants had similar effects (Gao et al., 2021b). There is a paucity of research on intercropping in relation to pests. Soybean-chrysanthemum intercropping was effective in limiting the incidence of the diurnal night moth, but the incidence of the tobacco aphid showed an increasing trend (Ratnadass et al., 2012).

Numerous works have indicated that microorganisms contribute significantly to soil nutrition and fertility (Zeng et al., 2019). Proper planting patterns help to keep soil microorganisms active and increases the variety of beneficial bacteria, thus changing the soil environment (Chaudhary et al., 2011; Tian et al., 2019). Unlike other common crops, tobacco can change the physicochemical properties of the subsurface through microorganisms in the soil, thus affecting different soil enzymes (Liu et al., 2018; Yang et al., 2019). Tobacco-marigold intercropping helps to improve soil nutrients and affect soil-associated bacteria, and is beneficial to reduce tobacco bacterial wilt (Li et al., 2020b). Moreover, Bai et al. (2022) conducted intercropping of Walnut and tea, which was found to obviously increase soil nutrient profile and enzymatic activity, as well as significantly improve plant health and growth by positive effects on microbial community. Li and Wu (2018) reported that intercropping was able to increase the species and numbers of bacteria and fungi, but did not have a significant effect on soil structure. Carbon accumulation, soil nutrients, enzymes, and microorganisms, and the productivity of crop were enhanced with intercropping of cluster bean and elephant foot yam (Ilakiya et al., 2023). Intriguingly, intercropping Sugarcane and Legume in greenhouse can increase sugarcane yields without deteriorating the quality of the sugarcane (Malviya et al., 2021). Consequently, soil properties, rhizosphere microbiota, and plant species should be taken into consideration for the intercropping.

**Figure 1:**
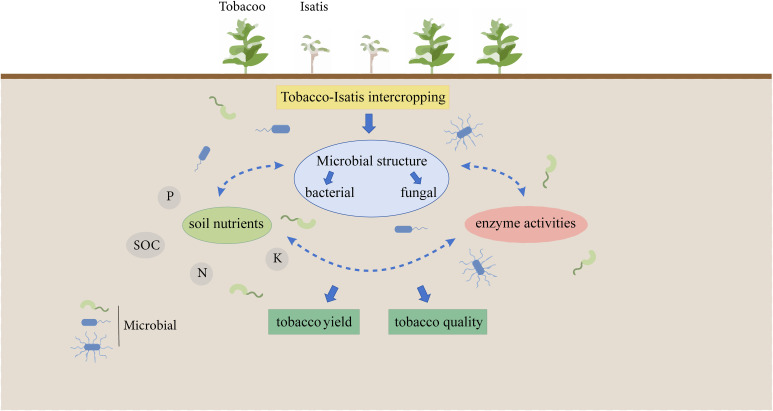
Schematic illustration of system integration from issues to outcomes.

Both roots and leaves of Isatis (*Isatis indigotica Fortune*), a genus of Isatis in the cruciferous family, are used as medicine, which are known respectively as Panax and Daphyllum. Intercropping Isatis with yellow Melilot effectively improved ecological performance (von Cossel et al., 2019). However, studies comparing the cropping performances and mechanisms of tobacco monocropping (B1) and different tobacco/Isatis intercropping (B2, B3, and B4) on the soil microorganisms are insufficient, which should be further clarified (**Figure 1**). Our aim was to: (і) Study the influence of B1, B2, B3, and B4 on soil nutrient status; (ii) evaluate the influence of B1, B2, B3, and B4 on soil microorganisms; (iii) search the relevance between tobacco soil nutrients and bacterial microorganisms. It is hoped that the new findings from this study would help in the further development of the application of intercropping to mitigate continuous cropping barriers in tobacco plants.

## 2 Materials and methods

### 2.1 Experimental treatments and sampling

The trials were done at the Qingya Village, Shijiaheqiaogou Village (36.31°N, 118.41°E), Sitou Town, Weifang City, Shandong Province, China and Tobacco Comprehensive Laboratory, Shandong Agricultural University in 2023.

**Figure 2:**
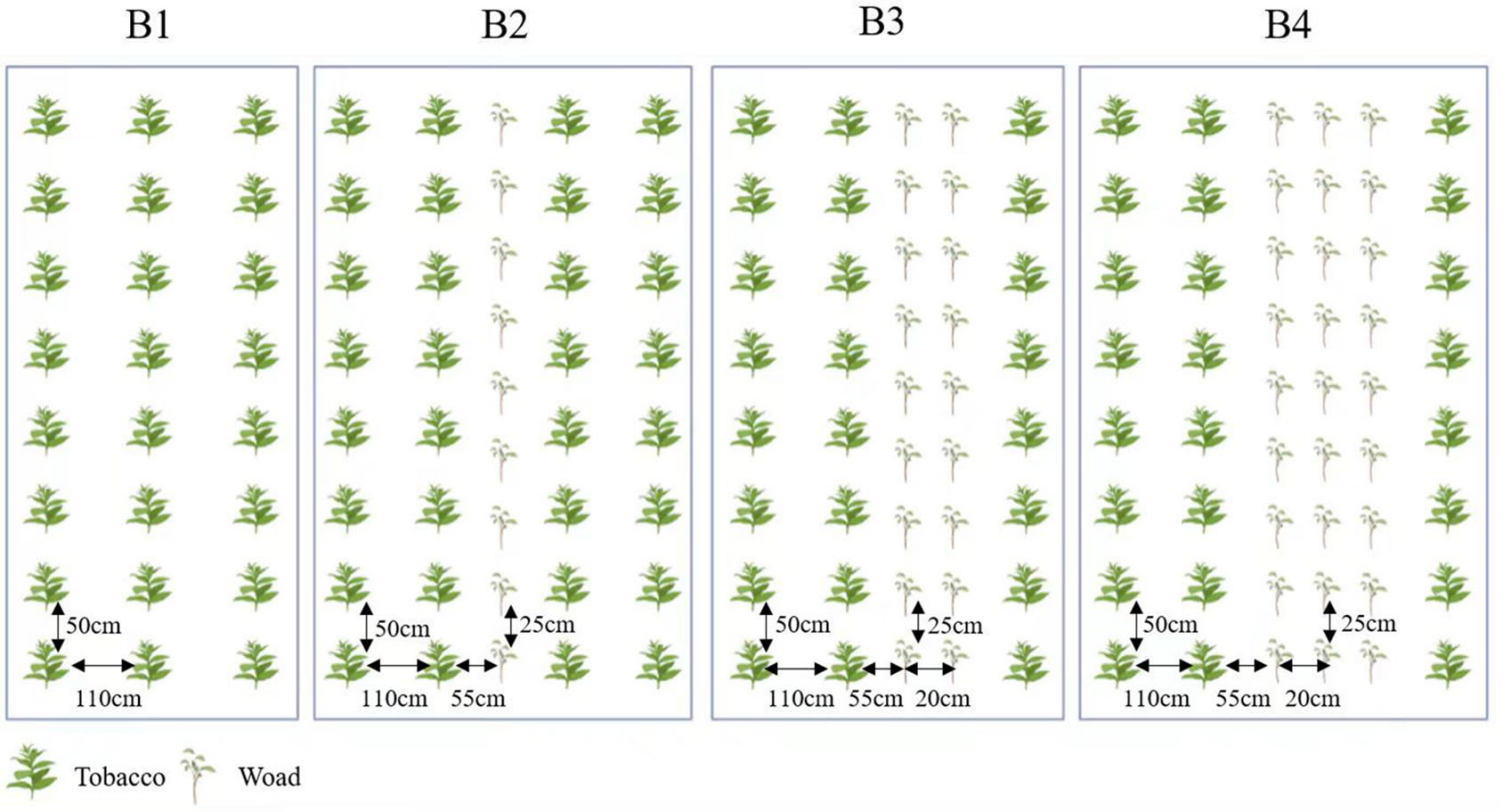
Planting system of tobacco and Isatis intercropping. B1, tobacco monocropping; B2, tobacco-Isatis intercropping, plant in rows at a ratio of 2:1 (tobacco: Isatis); B3, tobacco-Isatis intercropping, plant in rows at a ratio of 2:2 (tobacco: Isatis); B4, tobacco-Isatis intercropping, plant in rows at a ratio of 2:3 (tobacco: Isatis).

Our trial was divided into four treatments: (I) tobacco (cv. Yunyan87) monocrop, tobacco seedlings were planted at 1,200 plants per mu, with 110 cm apart in rows and 50 cm apart in plants; (II) plant in rows at a ratio of 2:1 (tobacco: Isatis), with two rows of tobacco and a row of Isatis were alternated, 110 cm apart in rows and 50 cm apart in plants. (III) plant in rows at a ratio of 2:2 (tobacco: Isatis), with two rows of tobacco and two rows of Isatis were alternated, 110 cm apart in rows and 50 cm apart in plants. (IV) plant in rows at a ratio of 2:3 (tobacco: Isatis), with two rows of tobacco and three rows of Isatis were alternated, 110 cm apart in rows and 50 cm apart in plants. In the intercropping pattern, rows of Isatis were spaced 20 cm apart and plant spacing was 25 cm, and rows of Isatis were spaced 55 cm apart from rows of tobacco (**Figure 2**).

On 10 September 2023, the soil samples (10-20 cm) were collected in the maturity period of tobacco by using the five-point sampling method. Soil samples for B1 were collected from between rows, and B2, B3, B4 were collected from between the rows of tobacco and Isatis. Each treatment was replicated three times, taking about 100 g of soil for each repeat, mixed thoroughly to filter out obvious impurities, and grouped into B1, B2, B3, and B4. Samples were sent to the laboratory without delay after collection. The retrieved samples were stored separately, partially dried naturally for one week and then passed through a 1 mm sieve for soil nutrient determination, partially refrigerated at 4°C for determination of the relevant enzyme activity and the remainder frozen at -80°C for 16S rRNA sequencing.

### 2.2 Soil nutrients analyses and enzyme activities

Soil pH was determined using a pH meter after the soil was dissolved in water. Total soil organic matter (SOC) was measured by the dichromate volumetric method (Gao et al., 2021a; Wang et al., 2021). The alkaline hydrolysis diffusion method was used to determine soil alkaline nitrogen (AN) (Tang et al., 2021). Soil available phosphorus (AP) was determined by NaHCO_3_-molybdenum antimony colorimetry and soil available potassium (AK) by NH_4_OAc-flame photometry (Bao, S. D., 1999). Soil sucrase (BC0240), urease (BC0120), hydrogen peroxidase (BC0100) and β-glucosidase (BC0160) were extracted separately using the Solarbio kits and the enzyme activities were measured using an ultraviolet spectrophotometer (Hitachi UV1900).

### 2.3 Soil DNA extraction, PCR amplification and sequencing

DNA concentration and purity were determined with a microplate reader (Gene Compang Limited, synergy HTX). Amplification was carried out according to the assay and the integrity of the PCR products was tested by agarose electrophoresis at a concentration of 1.8% (Bomei Fuxin Technology Co., Ltd., Beijing, China).

Bacteria-specific fragments were highly variable 16S rRNA gene V3-V4 regions with the 338F: 5’- ACTCCTACGGGAGGCAGCA-3’/806R: 5’- GGACTACHVGGGTW-TCTAAT-3’ primer sets for bacteria. Fungi-specific fragments were ITS rDNA gene ITS1 regions with the ITS1F: 5’-CTTGGTCATTTAGAGGAAGTAA-3’/ITS2: 5’-GCTGCGTTCTTCATCGATGC-3’ primer sets for fungi. All amplifications were conducted in 10 µL responses with 0.3 µL of each primer, 5 ng of template, 2 µL 2 mM dNTPs, 0.2 µL of KOD FX Neo, 0.5 µL of KOD FX Neo Buffer, and ddH_2_O supplemented to 10 µL. The PCR conditions for the 16S V3-V4 rRNA gene were an initial denaturation step at 95°C for 5 minutes, followed by 25 cycles of 95°C for 30 seconds, 50°C for 30 seconds and 72°C for 40 seconds, and a final extension step at 72°C for 7 minutes. Target regional PCR products were purified and evaluated on 1.8% agarose gels. The results were quantified using Image J software, 150 ng of each sample was mixed and pooled, and the mixed samples were purified before gel cutting using e.Z.N.A.TM Cycle-Pure Kit (omega) columns and recovered by gel cutting using the Monarch DNA Gel Recovery Kit. And finally performed on the Illumina novaseq6000 platform at Biomarker Technologies Co, LTD (Beijing, China).

### 2.4 Quality and production value measurement

Tobacco yield of each treatment was counted after harvesting and roasting, and Isatis yield of B2, B3, B4 was counted. The output of crop yield was calculated based on the yield of tobacco and Isatis following the prices of nearby market. Finally, the total output value of tobacco fields of each treatment were determined. Representative post-roasting tobacco samples (B2F, X2F) were selected for the determination of reducing sugar, total sugar, nicotine and total nitrogen content using a continuous flow analyzer with reference to the tobacco industry standard YC/T159-161-2002 Continuous Flow Method for the Chemical Composition of Tobacco and Tobacco Products, and for the determination of potassium content in the tobacco using atomic absorption spectrometry.

### 2.5 Statistical analysis

Microsoft Excel 2019 and Origin 2022 were used to handle data on soil nutrients and soil enzyme activities to obtain trend lines and error values for the raw data and graphing, and the data were analyzed for significance using SPSS Statistics 26.0 method.

Quality control was performed on the raw sequenced sequences, including low quality filtering and length filtering. The resulting high-quality sequences were also clustered and divided into OTUs. The species classification of a Feature was obtained from its sequence composition. On the basis of the results of the Feature analysis, the samples were analyzed taxonomically at every taxonomic level to obtain a community structure diagram and a species clustering heat map for each sample at the taxonomic level of the phylum and genus. Species diversity within single samples was analyzed by Alpha diversity, analyzing Shannon, and Ace indexes for every sample. R software (version3.6.1) (Team, R.C., 2020) was employed to investigate the abundance (Ace) (Chao and Lee, 1992) and diversity (Shannon) of microbial communities (Keylock, 2005). The similarities and differences in bacterial and fungal community composition between the four planting methods were studied and presented with Principal Coordinate Analysis (PCoA) based on Bray-Curtis distance. The analysis of similarities (ANOSIM) was used to further test whether the four groups of bacterial and fungal communities were significantly different. A network analysis based on spearman’s rank analysis was conducted in this study using the 50 most abundant phylum in the microbial community to explore the relationships of different species in multiple samples. The correlation between the soil bacterial communities was concluded by a direct strong and significant correlation (*ρ >* 0.6, *P* < 0.01) between their interrelationships. The correlation between microbial communities and soil environmental parameters was examined using redundancy analysis (RDA).

## 3 Results

### 3.1 Effect of intercropping on the soil nutrients and enzyme activities

The nutrient characteristics of the different soil samples are described in **Table 1**. Obviously, significant differences in soil physicochemical characteristics were found between B1, B2, B3 and B4 treatments. Soil pH proliferated significantly in B2, B3, B4 by 6.75%, 4.72%, and 5.53%, compared with those in B1. Specifically, B2 significantly increased soil SOC content from 13.12 g/kg to 15.40 g/kg. Soil AP content significantly in B4 by 2.77%, compared with those in B1. In addition, B3 and B4 treatment significantly increased available nitrogen from 118.72 mg/kg to 127.93 mg/kg and 124.18 mg/kg. It was worth noting that soil AN content was not increase in the treatments after intercropping.

**Table 1:** Effect of intercropping on the soil nutrients.

Treatment	pH	SOC *^a^* (g/kg)	AN *^b^* (mg/kg)	AP *^c^* (mg/kg)	AK *^d^* (mg/kg)
B1	7.41 ± 0.08_b_	13.12 ± 0.66_b_	16.57 ± 0.20_a_	6.48 ± 0.20_a_	118.72 ± 0.10_c_
B2	7.91 ± 0.11_a_	15.40 ± 0.13_a_	15.87 ± 0.88a_b_	6.01 ± 0.04a_b_	109.63 ± 0.55_d_
B3	7.76 ± 0.08_a_	13.88 ± 0.60_b_	15.28 ± 0.73_b_	5.50 ± 0.13_b_	127.93 ± 0.25_a_
B4	7.82 ± 0.04_a_	13.12 ± 0.13_b_	14.82 ± 0.53_b_	6.66 ± 0.07_a_	124.18 ± 0.24_b_

Different letters in the same row indicate the degree of difference between treatments (Duncan’s test, *P* < 0.05, *n* = 3). Numbers following plus or minus signs represent standard deviations. B1, tobacco monocropping; B2, tobacco-Isatis intercropping (2:1); B3, tobacco-Isatis intercropping (2:2); B4, tobacco-Isatis intercropping (2:3). As follows. ^*a*^SOC, soil organic carbon; ^*b*^AN, available nitrogen; ^*c*^AP, available phosphorus; ^*d*^AK, available potassium. Different letters indicated the significant differences between treatments according to Duncan’s Multiple Range Test (DMRT) at P < 0.05.

Different letters in the same row indicate the degree of difference between treatments (Duncan’s test, *P* < 0.05, *n* = 3). Numbers following plus or minus signs represent standard deviations. B1, tobacco monocropping; B2, tobacco-Isatis intercropping (2:1); B3, tobacco-Isatis intercropping (2:2); B4, tobacco-Isatis intercropping (2:3). As follows.

### 3.2 Effect of intercropping on soil bacterial and fungal community diversity and structure

**Figure 3:**
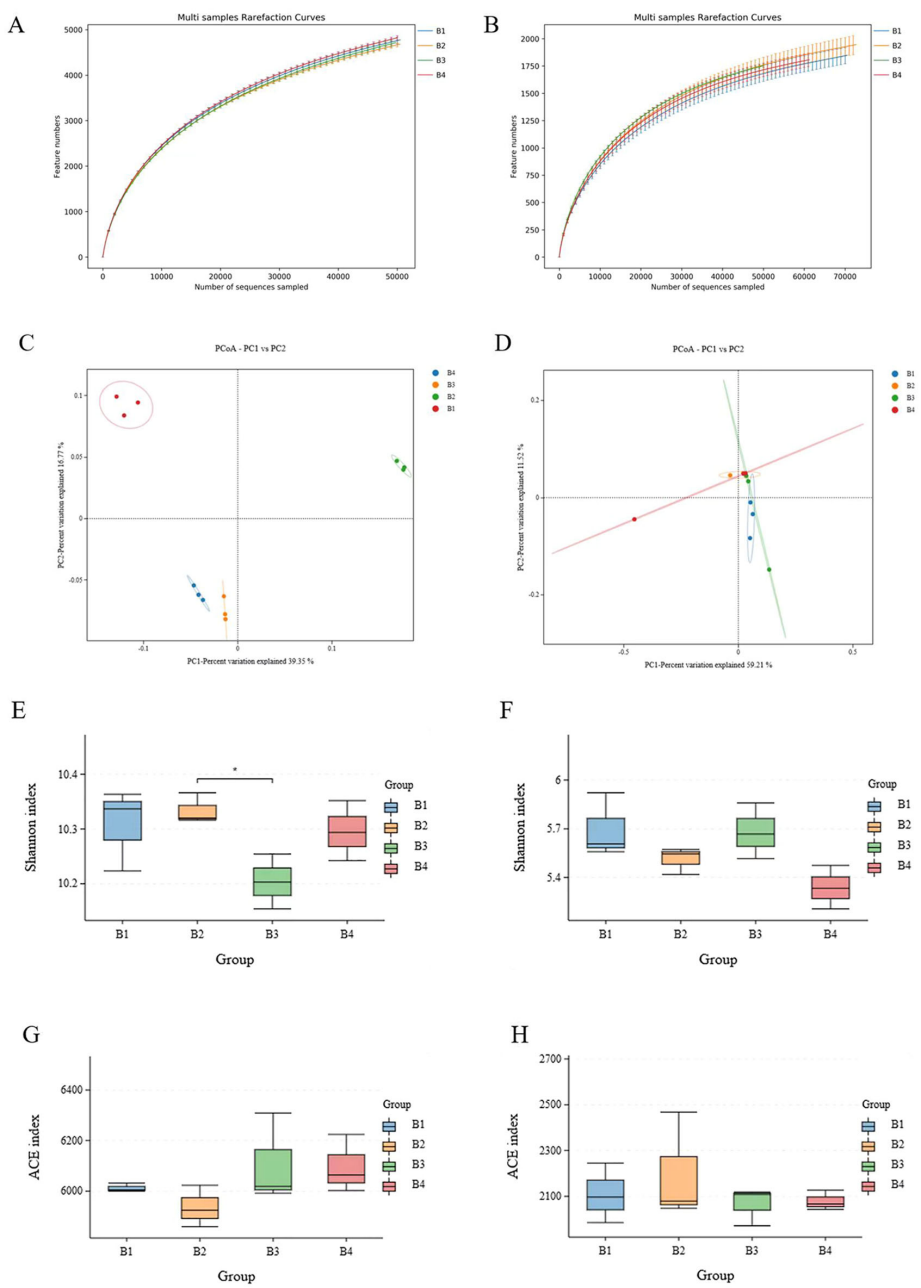
Species plots displaying the availability of bacterial (16S) **(A)** and fungal (ITS) **(B)** samples under B1, B2, B3 and B4. PCoA and Bray-Curtis distances indicating similarity between different treatments of bacteria **(C)** and fungi **(D)**. Shannon reflects the diversity of different processing bacterial **(E)** and fungal **(F)** species, ACE reflects the dominance of different processing bacterial **(G)** and fungal **(H)** species. B1, tobacco monocropping; B2, tobacco-Isatis intercropping (2:1); B3, tobacco-Isatis intercropping (2:2); B4, tobacco-Isatis intercropping (2:3). As follows. Asterisks indicated significant differences according to student’s t-test (*P < 0.05).

The different treatment soils were sequenced and classified as OTUs. The rarefaction curves showed clear asymptotes, proving that the sampling was nearly complete (**Figures 3A, B**). The results showed that there was a total of 7,207 bacterial OTUs in soil samples from the tobacco monoculture and 7,334 bacterial OTUs in soil samples from the B4, which were not significantly differed. The Shannon index of the soil bacterial communities was significantly different between B2 and B3 (**Figure 3E**), while differences in ACE diversity index between the four treatment groups were not significant (**Figure 3H**). There was a total of 3,231 fungal OTUs in soil samples from B1, 3,477 fungal OTUs in soil samples from the B2, 3,290 fungal OTUs in soil samples from the B3 and 3,885 fungal OTUs in soil samples from the B4. The Shannon index and ACE diversity index of the soil fungal communities were not significantly different among the four treatment groups (**Figures 3F, H**).

PCoA analysis demonstrated the whole similarity of bacterial and fungal community structure across different groups of soils by the different OTUs of the sequencing results. PCo1 accounted for 39.35% of the change in bacterial community composition, while PCo2 accounted for 16.77% (**Figure 3C**). PCo1 accounted for 59.21% of the change in fungal community composition, while PCo2 accounted for 11.52% (**Figure 3D**). PCoA analysis indicated different quadrants of soil bacterial distribution under different groups. Although there was a small overlap in the fungal communities of the treatments, there was a separation between the treatments. It was obvious that the separation between the samples was significant because of the different treatments, indicating significant differences in microbial structure in monocropping and intercropping systems. Anosim provided even stronger evidence that microbial community composition varied significantly depending on cropping patterns.

### 3.3 Effect of intercropping on soil bacterial and fungal composition

**Figure 4:**
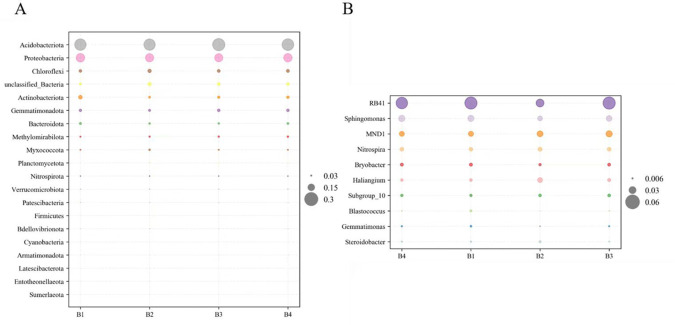
Composition of bacterial community in soil at phyla level **(A)**. Bacterial community composition at genus level **(B)**.

The bacteria of the soil samples at the phylum level are shown in **Figure 4A**. Acidobacteriota was the richest phylum, followed by Proteobacteria, Chloroflexi, Actinobacteriota and Gemmatimonadota (**Figure 4A**). It is noticeable that Chloroflexi (7-9%), Planctomycetota (2%), Nitrospirae (2%), and Verrucomicrobiota (2%) were significantly more abundant in B2 than those in B1. Moreover, Acidobacteriota (29-31%), Chloroflexi (7-9%), Gemmatimonadota (6-7%), Planctomycetota (2%), Nitrospirota (2%), and Verrucomicrobiota (2%) significantly more abundant in B3 and B4 than those in B1. In addition, a comparison of soil bacterial communities among different treatments was found at the genus level (**Figure 4B**). The richest genera in different treatments were *RB41* (4-6%), *Sphingomonas* (2-3%), *MND1*(2-3%), *Nitrospira* (2%), *Haliangium* (1-2%), and *Subgroup-10* (1%). *MND1*, *Nitrospira*, and *Subgroup-10* were significantly higher in B3 than in B1. *Nitrospira* and *RB41* were significantly higher in B4 than in B1.

**Figure 5:**
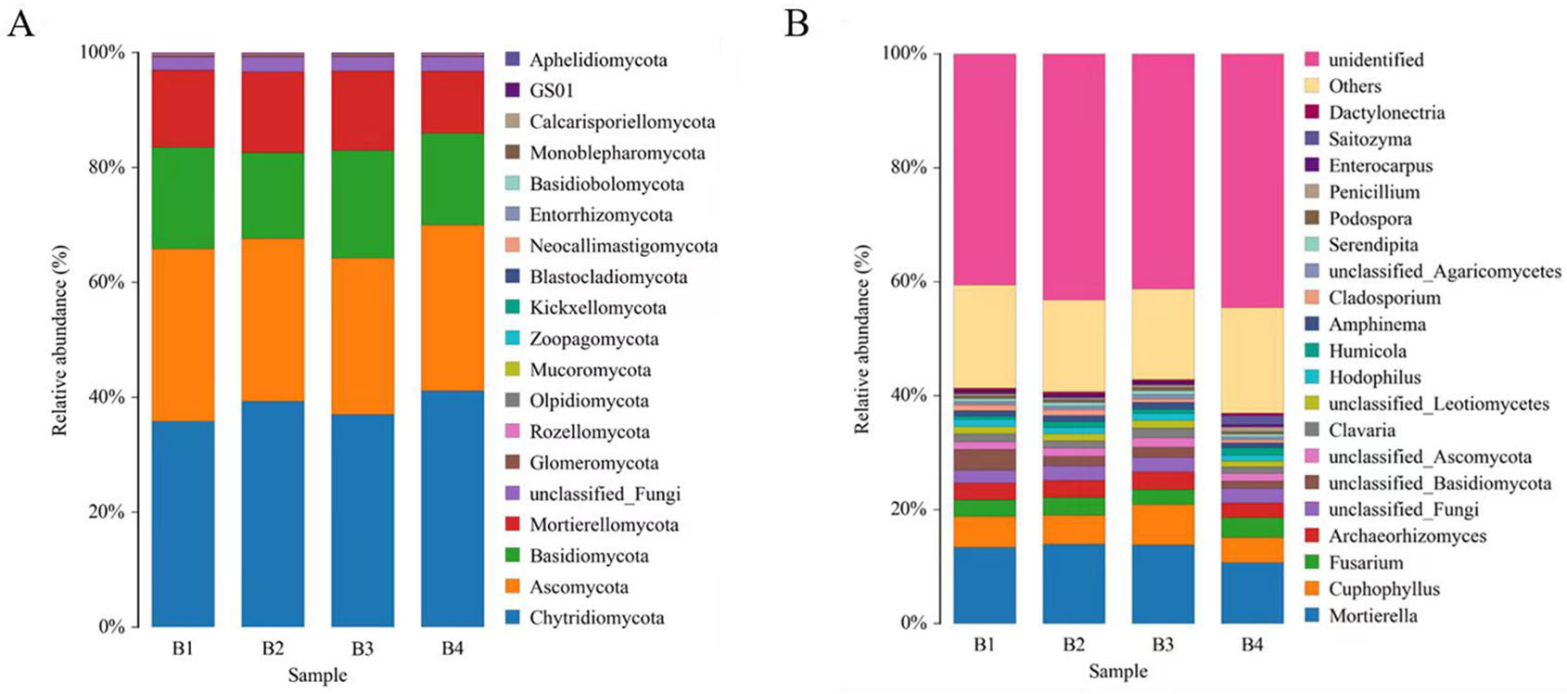
Composition of fungal community in soil at phyla level **(A)**. Bacterial community composition at genus level **(B)**.

As showed in **Figure 5**, tobacco-Isatis intercropping changed the community composition and relative abundance of fungi in the soil based on phylum and genus levels. The fungi with the highest relative abundance at the phylum level were Chytridiomycota (36-41%), Ascomycota (27-30%), Basidiomycota (15-19%), and Mortierellomycota (11-14%). Compared with the B1, Ascomycota and Basidiomycota were lower abundance in B2 and B4, Ascomycota were lower abundance in B3 and Mortierellomycota were lower abundance in B2 and B3 (**Figure 5A**). The richest genera in different treatments were *Mortierella* (11-14%), *Fusarium* (3%), *Archaeorhizomyces* (3%), and *Clavaria* (1-2%). *Mortierella* was significantly higher in B2 and B3 than in B1. Compared with the B1, *Fusarium* was significantly lower in B3 and *Archaeorhizomyces* was significantly lower in B4.

### 3.4 Network analysis of soil bacterial and fungal communities

**Figure 6:**
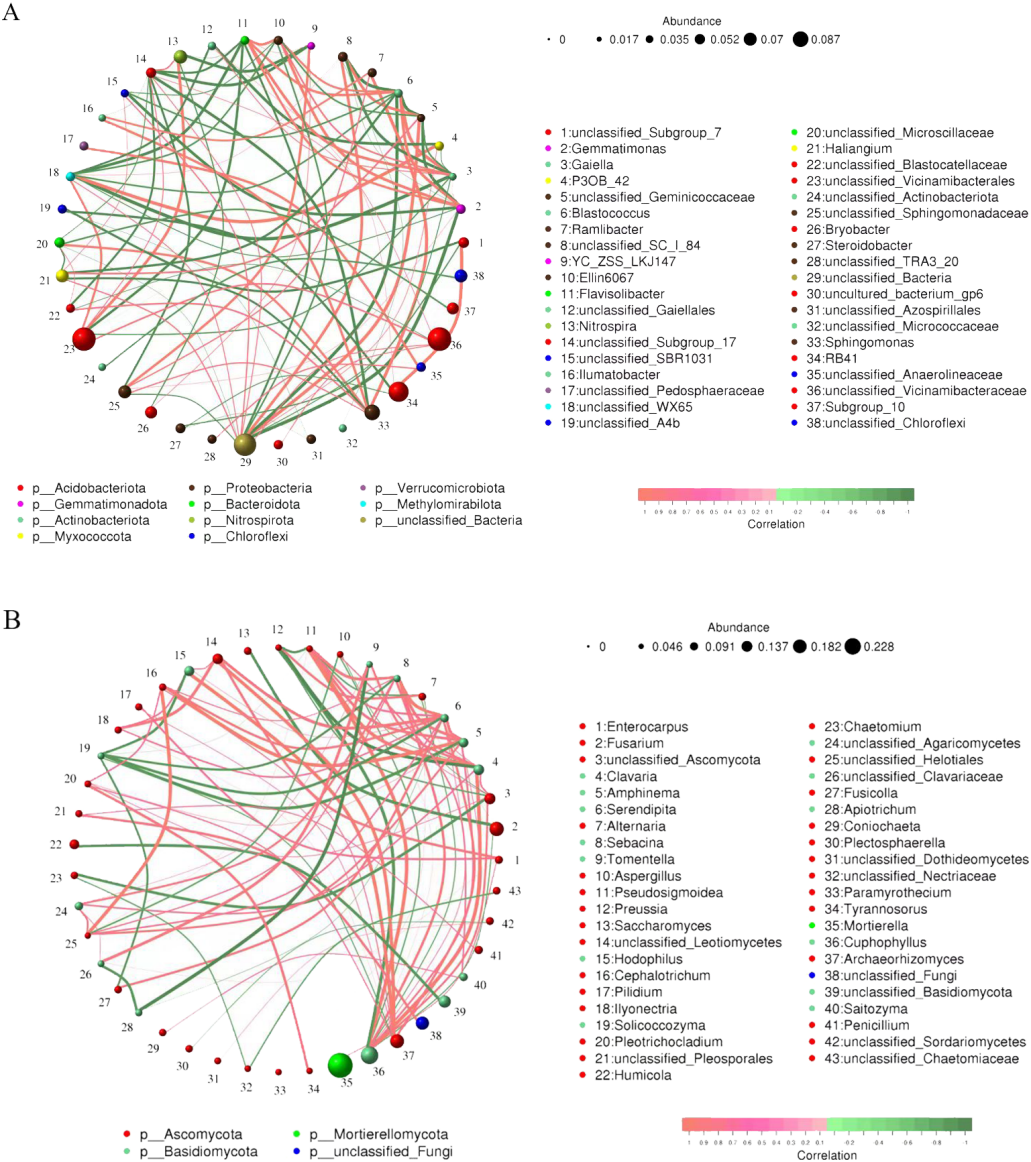
Network of symbiotic bacterial **(A)** and fungal **(B)** genera in tobacco-Isatis intercropping based on correlation analysis. The line between the points represents a significant correlation (Spearman’ s *ρ* > 0.6, *P* < 0.01). The size of the node represents the abundance of bacteria and the width of the connecting line represents the Spearman’s correlation coefficient value. The red line indicates a significant positive relationship between two nodes, while the green line indicates a significant opposite relationship.

To investigate the symbiotic nature and potential key taxa of microorganisms in soil samples, a network analysis was performed based on the top 50 abundant genera. These networks demonstrated significant differences in their structure and characteristics (**Figure 6**). The soil bacterial community consisted of 38 nodes and the soil fungal community consisted of 43 nodes, and the positive correlation was higher than the negative correlation for both communities. Bacterial community had 47% of the positive links, while fungal community accounted for 63%. It’s worth noting that bacterial network complex is higher compared with fungal communities. In the network of bacterial communities, *Gaiella*, *Blastococcus*, *Flavisolibacter*, and *Gemmatimonas* occupied more nodes. In the fungal community, *Serendipita*, *Clavaria*, *Cuphophyllus*, and *Sebacina* occupied more nodes. The above results indicated that the bacteria in the tobacco/Isatis intercropping soil developed a highly interactive and complicated network. As with other community analyses, the Proteobacteria and Acidobacteria phyla appeared to take a vital part in establishing the social nature of the microbiota.

### 3.5 Relationships between soil nutrients, soil enemy activities and soil bacterial and fungal communities

**Figure 7:**
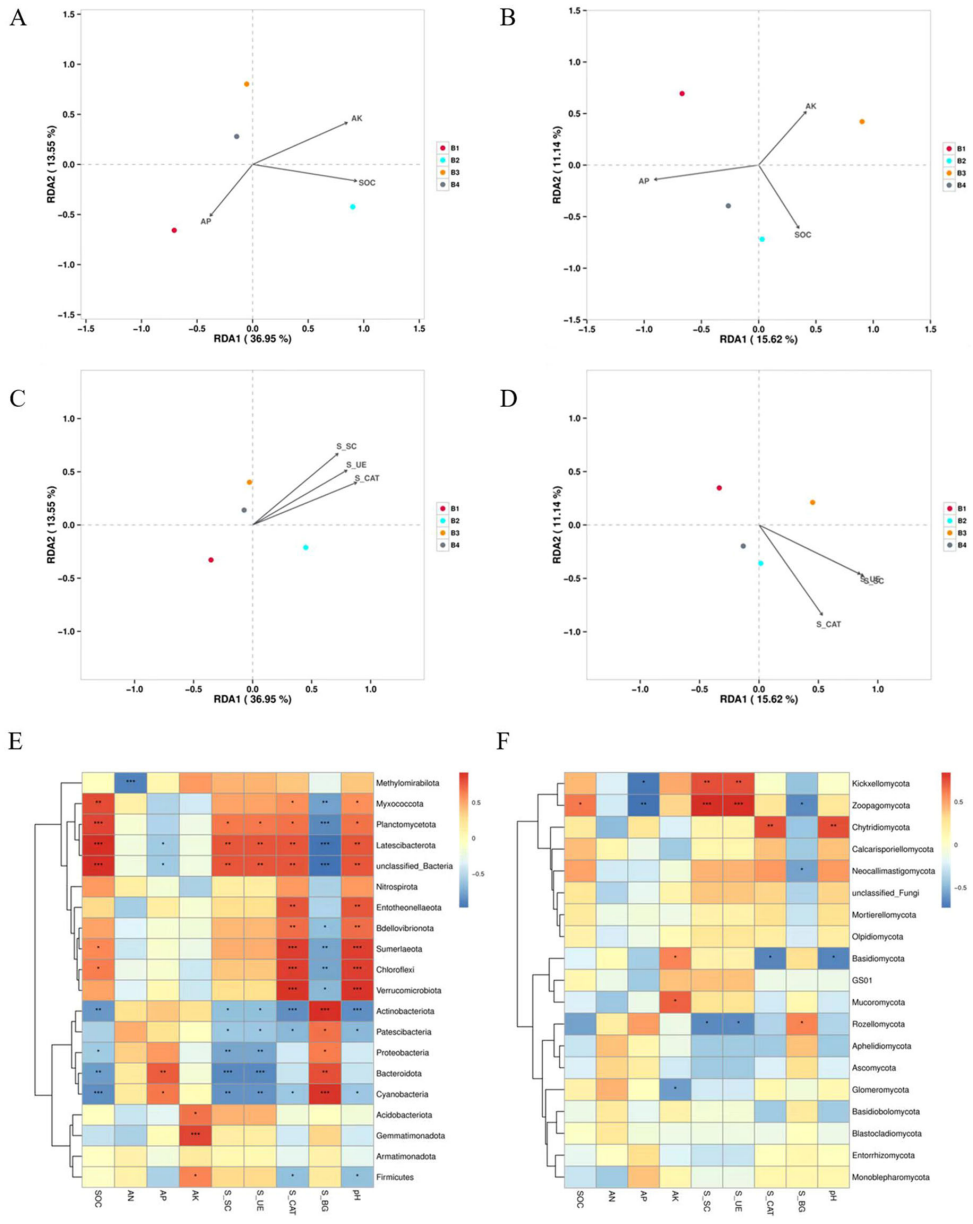
Correlation between bacteria and nutrients **(A)**, enzyme activities **(C)** in soil samples and correlation heatmap between nutrients, soil enzyme activities and dominant bacteria in samples **(E)** correlation between fungi and nutrients **(B)**, enzyme activities **(D)** in soil samples and correlation heatmap between nutrients, soil enzyme activities and dominant fungi in samples **(F)**. S_SC, Sucrase; S_UE, Urease; S_CAT, Catalase, S_BG, β-glucosidase. *0.01 < *P* ≤ 0.05, **0.001 < *P* ≤ 0.01, ***P ≤ 0.001.

The influence of soil nutrients and soil enemy activities on soil microorganisms was analyzed by redundancy analysis (RDA). As shown in **Figure 7**, it was clearly evident that there were clear differences in the composition of bacteria and fungi under different treatments. The soil SOC, AP, and AK all were actively related to the microbial communities in intercropping system. AP was positively correlated with bacterial community changes in B1 and negatively correlated with B2, B3, and B4. SOC was positively correlated with bacterial community changes in B2 and negatively correlated with B1, B3, and B4. AK was positively correlated with bacterial community changes in B2, B3, and B4 and negatively correlated with B1 (**Figure 7A**). AP was positively correlated with changes in fungal communities in B1, B2, and B4, and negatively correlated with B3. SOC was positively correlated with changes in fungal communities in B2, B3, and B4 treatments, and negatively correlated with B1. AK was positively correlated with changes in fungal communities in B1, B3 treatments, and negatively correlated with B2 and B4 (**Figure 7B**). In terms of soil enzyme activities of the different treatments, three enzyme activities were positively associated with changes in microbial communities in the three intercropping treatments (**Figures 7C, D**).

Planctomycetota were strongly associated with the relative abundances of SOC, S_SC, S_UE, S_CAT (**Figure 7E**). Chloroflexi and Verrucomicrobiota displayed a significant active association with soil SOC and S_CAT. Acidobacteria and Gemmatimonadota were positively associated with AK. Noticeably, Actinobacteria and Cyanoacteria were positively correlated with soil S_BG, whereas they were negatively correlated with SOC, S_SC, S_UE, S_CAT, and pH. Basidiomycota showed a negative correlation with pH and S_CAT, and Kickxellomycota and Zoopagomycota were negatively correlated with AP (**Figure 7F**).

### 3.6 Effect of intercropping on tobacco yield and production value

As shown in **Table 2**, the B1 had obviously high tobacco yield of 173.31 kg per mu. After intercropping with Isatis, the planting area of tobacco decreased, which was the main reason for the reduction of the tobacco yield and output value benefit. Compared with tobacco monocropping, the average price of the B2 was slightly lower, and the average price of the tobacco was significantly higher in all cases of intercropping between tobacco-Isatis intercropping. The average price of B2 was 3.88 dollars, which increased by 1.03%, the average price of B3 was 3.91 dollars, which increased by 1.83%, and the average price of B4 was 3.98 dollars, which increased by 3.58%.

**Table 2:** Effect of intercropping on the output value of tobacco.

Treatment	Output (kg/667 m^2^)	Average Price (USD/kg)	Output value benefit (USD/667 m^2^)
B1	173.31_a_	3.84_d_	665.96_a_
B2	164.90_b_	3.88_c_	640.23_c_
B3	163.41_c_	3.91_b_	639.28_d_
B4	160.81_d_	3.98_a_	639.96_b_

Different letters indicated the significant differences between treatments according to Duncan’s Multiple Range Test (DMRT) at P < 0.05.

As shown in **Table 3**, tobacco-Isatis intercropping can also increase the output value benefits through both tobacco and Isatis. The total output value of the tobacco field in B2 was 669.00 dollars, B3 was 697.05 dollars, and B4 was 726.62 dollars, which were higher than B1. The total output value of the tobacco field in intercropping of tobacco-Isatis intercropping was increased compared with tobacco monocropping.

**Table 3:** Effect of intercropping on the output value of Isatis.

Treatment	Output (kg/667 m^2^)	Average Price (USD/kg)	Output value benefit (USD/667 m2)	Total output value of tobacco fields (USD/667 m2)
B1	/	/	/	665.88_d_
B2	17.48_c_	1.65_a_	28.89_c_	669.04_c_
B3	34.96_b_	1.65_a_	57.77_b_	697.07_b_
B4	52.44_a_	1.65_a_	86.66_a_	726.63_a_

Different letters indicated the significant differences between treatments according to Duncan’s Multiple Range Test (DMRT) at P < 0.05.

### 3.7 Effect of intercropping on tobacco quality

As shown in **Table 4**, all three treatments of tobacco-Isatis intercropping showed significant increase in the proportion of superior smoke after roasting. B1 had the lowest proportion of superior tobacco at 53.34%. B2 accounted for 57.71%, which was 8.19% higher than that of B1; B3 accounted for 62.27%, which was 16.74% higher than that of B1; and B4 accounted for 67.91%, which was 27.32% higher than that of B1. The results showed that tobacco-Isatis intercropping could increase the proportion of top-grade tobacco after roasting and effectively improve the quality of tobacco.

**Table 4:** Effects of intercropping between tobacco and Isatis on the proportion of upper-grade tobacco.

Treatment	Proportion of upper tobacco(%)
B1	53.34_c_
B2	57.71_bc_
B3	62.27_ab_
B4	67.91_a_

Different letters indicated the significant differences between treatments according to Duncan’s Multiple Range Test (DMRT) at P < 0.05.

As shown in **Table 5**, the content of reducing sugar, total sugar, nicotine, potassium and two sugar ratio were increased in two parts of leaves in all treatments after tobacco-Isatis intercropping. The chemical composition of tobacco leaves in the upper leaves (B2F) of all treatments were within the suitable range, and the differences were significant between monocropping and intercropping treatments. The total sugar content of central leaf (C3F) of B1, B2, and B3 treatments were 24.05%, 25.73%, and 24.27%, respectively, which were beyond the suitable range of total sugar (18-24%), and the sucking flavor of tobacco was bland when total sugar content was too high, the total sugar content of B4 was 23.97%, which indicated that the intercropping could make the total sugar content be controlled in the suitable range. The nicotine content of the central leaf of B4 treatment was 3.80%, which exceeded the appropriate range (1.5-3.5%), and the high nicotine content would make the smoking taste of tobacco too heavy. The potassium content of the central leaf of B1, B2, and B3 treatments was slightly lower than the appropriate range of 2%, while the potassium content of the B4 treatment was 2.09%, which indicated that intercropping of roasted tobacco and woad could appropriately increase the potassium content of the tobacco and increase the eating taste. As a very important index in the evaluation of tobacco quality, sugar-alkali ratio is in the range of 6-10, and the sugar-alkali ratio in the middle leaf of B1 is 10.75, which is beyond the suitable range and will increase the irritation of the smoke and flavor. The sugar-alkali ratio of the intercropping treatments were all within the normal range, which indicated that tobacco-Isatis intercropping could regulate the sugar-alkali ratio of the tobacco and improve the quality of the tobacco.

**Table 5:** Effects of intercropping between tobacco and Isatis indigotica on the chemical composition of tobacco leaves.

Treatment	Grade	Reducing sugars (%)	Total sugars (%)	Total nitrogen (%)	Nicotine (%)	K (%)	Glycemic ratio	Total sugar to reducing sugar ratio	Ratio of nitrogen to alkali
B1	B2F	16.58_d_	21.64_b_	2.06_c_	2.23_c_	1.45_c_	9.68_a_	0.77_c_	0.92_c_
B2		17.88_c_	22.03_a_	2.11_b_	2.25_b_	1.97_a_	9.77_a_	0.81_b_	0.94_b_
B3		20.51_a_	22.07_a_	2.18_a_	2.27_a_	1.86_b_	9.73_a_	0.93_a_	0.96_a_
B4		19.11_b_	21.33_c_	2.09_b_	2.23_c_	1.89_b_	9.55_b_	0.90_a_	0.94_b_
B1	C3F	18.30_b_	24.05_c_	2.05_b_	2.24_d_	1.74_c_	10.75_a_	0.76_b_	0.91_a_
B2		18.46_b_	25.73_a_	2.11_a_	3.06_c_	1.61_d_	8.40_b_	0.72_c_	0.69_b_
B3		19.10_a_	24.27_b_	2.15_a_	3.15_b_	1.99_b_	7.70_c_	0.79_a_	0.68_b_
B4		16.42_c_	23.97_c_	2.11_a_	3.80_a_	2.09_a_	6.30_d_	0.69_d_	0.56_c_

Different letters indicated the significant differences between treatments according to Duncan’s Multiple Range Test (DMRT) at P < 0.05.

## 4 Discussion

A number of studies have indicated that numerous properties of the soil can change due to different cropping patterns (Tang et al., 2020; Zhao et al., 2023). When there is sufficient AN in the soil, tobacco can synthetic more protein, promote cell division and cell growth, and increase photosynthesis. As an important indicator, AN in soil closely influences the dynamics of the soil nitrogen pool, and its level and dynamics can directly affect soil fertility (Hu et al., 2018). This research showed that soil available nitrogen was slightly reduced in B2, B3 and B4 (**Table 1**). Although diversified plots should begin to have greater potential to contribute to increased nitrogen and improved soil fertility, the long history of unsustainable management of these soils, high rates of erosion, and lack of vegetative cover make them extremely sensitive to management disturbances and degradation (Aguilera-Huertas et al., 2024). In this study, it probably due to the imbalance of soil nutrient ratios and deterioration of soil quality caused by the perennial continuous cropping of tobacco, which affected the uptake of available nitrogen by tobacco. It is generally accepted that due to the reabsorption and use of nitrogen nutrients that could not be absorbed and utilized in tobacco fields, the over-absorption of nutrients by tobacco plants was limited and reduced, and the supply of nutrients was more balanced (Edwards et al., 2017). Soil organic matter content is an essential index used to measure soil fertility. The introduction of intercropping, reduced tillage and increased crop residues can be effective in increasing soil organic matter, soil fertility and biodiversity, while increasing crop yields and reducing fertilizer use (Marcos-Pérez et al., 2023; Roohi et al., 2022). In this study, soil organic matter content was significantly higher in B2 (**Table 1**), indicating that compared with single cropping, intercropping significantly improved soil fertility. Relevant studies in recent years have also mentioned that AP and AK contents of B4 was increased significantly compared with B1 (**Table 1**). Soil available phosphorus helps to enhance the resistance of tobacco to disease, drought and cold, and it is very important for the quality of tobacco (Sonmez et al., 2016). The increase in soil phosphorus fertility is mainly due to a decrease in soil adsorption of phosphorus and a decrease in insoluble phosphorus complexes in the soil. In the case of organic phosphorus pools, this is mainly regulated by soil microbiological processes (Roohi et al., 2020). Soil AK in the soil promotes the synthesis and operation of carbohydrates, increases the efficiency of light and systems, facilitates normal plant development and increases the resistance of plants to pests and drought, and promotes the metabolism of nitrogen in tobacco.

Soil enzyme activity represents the extent of soil nutrient cycling (Nannipieri et al., 2012; Zhou et al., 2019). Sucrase promotes the accumulation of labile nutrients in the soil environment, and the stronger the soil sucrase, the higher the soil fertility. Sucrase determines to a certain extent the degree of soil maturation and soil fertility, and determines the intensity of soil biological activity (Liu et al., 2014). It is clear from the experiment that sucrase activity increased and soil fertility was enhanced after intercropping (**Table 6**). Soil urease enhances the content of nitrogenous and carbonaceous compounds in the soil environment and promotes the hydrolysis of urea, and the level of which has an important influence on the abundance of microbial organic matter. Urease also represents the level of soil nitrogen content and can effectively promotes the transformation of SOC and regulates the efficiency of energy metabolism. Similarly, the increase of soil urease activity indicates the improvement of soil fertility, and intercropping clearly enhances soil fertility. Consistent with the results of this experiment, the higher the utilization of inorganic nitrogen, the lower the peroxidase activity (Cantarella et al., 2018). Therefore, we speculate that tobacco-Isatis intercropping can be hypothesized to improve soil fertility and transfer nitrogen to the soil. Previous studies have reported that soil enzyme activities were positively correlated with soil organic carbon and nitrogen contents. Higher organic matter and nitrogen content in intercropping systems may be another reason for the increase in soil enzyme activities (Wang et al., 2024).

**Table 6:** Effect of intercropping on the soil enzyme activities.

Treatment	Sucrase (U/g)	Urease (U/g)	Catalase (U/g)	β-glucosidase (U/g)
B1	4.82 ± 0.48_d_	1089.82 ± 15.89_c_	0.38 ± 0.02_c_	11.45 ± 0.8_a_
B2	16.19 ± 0.55_b_	4537.66 ± 51.96_a_	2.08 ± 0.07_a_	6.04 ± 0.47_c_
B3	18.03 ± 0.48_a_	4596.18 ± 46.43_a_	1.56 ± 0.14_b_	8.38 ± 0.24_b_
B4	12.31 ± 0.17_c_	2512.21 ± 33.27_b_	1.66 ± 0.19_b_	9.25 ± 0.42_b_

Different letters indicated the significant differences between treatments according to Duncan’s Multiple Range Test (DMRT) at P < 0.05.

High levels of soil microbial alpha diversity indices help to maintain the sustainability and increase the resistance of soil ecosystems and ensure their proper functioning (Li et al., 2020a). ACE index is an important indicator of species dominance in soil bacterial communities, and the Shannon index reflects the diversity of the soil bacterial community (Fan et al., 2022). Some researchers did not find significant changes in the alpha index (Fu et al., 2019), and they did not draw firm conclusions about whether intercropping affected the alpha index. And no significant differences were found between the Shannon and ACE indices of two treatments (**Figures 3E–H**), which is consistent with their findings.

The increase in soil microbial abundance can lead to enhanced activity of functional microorganisms (Weller et al., 2002). Isatis is a medicinal Chinese herb that may secrete nutrients and exudates following intercropping with tobacco, which may affect the microbial community of B2, B3 and B4. Compared with monocropping, the increase in bacterial OTUs for B4 seems to indicate this. The main bacterial taxa found in the soils tested were Acidobacteriota, Proteobacteria, Chloroflexi, Actinobacteriota, and Gemmatimonadota, all of these have been described as frequent bacteria of the soil. A higher relative abundance of Acidobacteria, Chloroflexi, Gemmatimonadota, Planctomycetota, Nitrospirae, and Verrucomicrobiota in B3 and B4 than in B1 indicated that cropping patterns can alter the abundance of major bacterial phyla as they adjust to new microenvironment (**Figure 4C**). Actinobacteria can enhance host nutrient acquisition and are famous for their beneficial influences on plants (Palaniyandi et al., 2013; Shi et al., 2019). Furthermore, Bacteroidota has been identified as being related to the cycling of soil nitrogen and phosphorus (Lidbury et al., 2021), Gemmatimonadota could reduce the proportion of harmful fungi (Solis et al., 2020). Acidobacteriota also has an important part in the cycling process. And numerous bacteria have been identified as beneficial to plants and can promote plant growth. All three intercropping treatments showed a reduction in the abundance of the pathogenic fungus Ascomycota compared with the B1, and a study with *Pandanus amaryllifolius* and *Areca Catechu* intercropping also demonstrated a reduction in the abundance of Ascomycota after intercropping (Zhong et al., 2022).

Numerous researches have indicated that intercropping patterns have an essential role to the composition of microbial communities (Tian et al., 2019; Rezaei-Chiyaneh et al., 2021). By combining microbial composition with the soil chemical properties through RDA, we found that soil nutrients and soil enemy activities were positively linked to soil microbial community changes (**Figures 7A–D**), indicating that intercropping system had a significant influence on the soil microbial communities. In addition, environmental indexes such as soil SOC, AP, and AK all accelerated changes in the microbial community, which confirmed the findings of Pang et al (Pang et al., 2022).

Plant microbes do not function as a sum of their individual parts, as microbial communities can react with others to make for a rich network (van der Heijden and Hartmann, 2016). There was a difference in the number of nodes in the bacterial and fungal network systems, and from bacteria and fungi accounted for 47% and 63% of the total links (**Figure 6**), suggesting that tobacco-Isatis intercropping systems caused more competitive correlations between microorganisms to be induced. The results showed that the interactions between soil bacteria and fungi were extremely complex, which was in accordance with Li et al. (Li and Wu, 2018). The more complicated symbiotic network of the intercropping treatment suggested an ecosystem function with enhanced soil carbon and nutrient cycling (Yan et al., 2021).

The chemical composition of tobacco leaves was increased in intercropping treatments indicated the potential role of intercropping on plant production. The improvement of tobacco yield and quality in intercropping may be related to the ability to regulate the interactions of the underground parts of the crop, as microbiologically rich soils have higher organic matter decomposition and plant productivity (Delgado-Baquerizo et al., 2020; Lan et al., 2023). Tobacco-Isatis intercropping can increase the abundance of beneficial bacteria in the soil, reduce the abundance of harmful fungi, accelerate the release of enzymes in the soil, accelerate the decomposition of organic matter, improve the growth environment of the tobacco plant, and promote the growth and development of tobacco.

## 5 Conclusion

Intercropping had a relatively large impact on the changes of soil microorganisms, which correlated with environmental indexes such as SOC, pH and AN. Intercropping tobacco with Isatis had an impact on the diversity and dominance of the soil bacterial community compared with tobacco monocropping. The increase of some beneficial microorganisms, such as Bacteroidota, Gemmatimonadota, and Acidobacteriota had positive effects on tobacco growth and development, providing an effective aid to pest and disease mitigation. This study showed that tobacco and Isatis intercropping improved soil nutrients, soil enzyme activities, optimized soil bacterial community structure, improved tobacco leaf quality and increased tobacco yield and output value. However, it is one-sided to look only at the changes in soil microbial community abundance by the four treatments in this study, and more time is needed to demonstrate whether these practices have a potentially positive impact on soil microbial structure and improved soil quality. Therefore, long-term monitoring programs are needed to provide reliable data on these improvements, as soil change is a long-term process.

## Data Availability

The original contributions presented in the study are included in the article/supplementary material. Further inquiries can be directed to the corresponding authors.
